# Acupuncture combined with Tai Chi in the treatment of a patient with coccyx fracture: A case report

**DOI:** 10.1097/MD.0000000000044068

**Published:** 2025-08-29

**Authors:** Xue Bai, Yukui Tian, Cheng Wang, Jingxian Li, Lingjun Kong, Lei Guo, Junchang Liu, Qingguang Zhu

**Affiliations:** a Department of Acupuncture and Moxibustion and Massage, College of Traditional Chinese Medicine, Xinjiang Medical University, Urumqi, China; b Department of Tuina, Fourth Clinical Medical College, Xinjiang Medical University, Urumqi, China; c School of Human Kinetics, Faculty of Health Sciences, University of Ottawa, Ottawa, Canada; d Department of Massage, Affiliated Shuguang Hospital, Shanghai University of Traditional Chinese Medicine, Shanghai, China; e Department of Physical Therapy with Chinese Massage, Yueyang Hospital of Integrated Traditional Chinese and Western Medicine, Shanghai University of Traditional Chinese Medicine, Shanghai, China.

**Keywords:** acupuncture, coccyx fracture, subluxation, Tai Chi, traditional Chinese medicine

## Abstract

**Rationale:**

Coccyx fractures and subluxations are commonly caused by trauma, often leading to severe pain and restricted mobility. Traditional treatments focus on analgesia and immobilization; however, recovery periods are prolonged. This study explores the efficacy of acupuncture combined with Tai Chi in promoting rehabilitation for coccyx fractures.

**Patient concerns:**

A 34-year-old female patient experienced sacrococcygeal pain and limited mobility after falling on her buttocks while skiing on February 13, 2025.

**Diagnoses:**

Computed tomography imaging indicated a suspected fracture of the S5 vertebral body, a possible subluxation of the Co1 vertebral body, and soft tissue swelling with effusion in the sacrococcygeal region.

**Interventions:**

The treatment consisted of lumbosacral acupuncture, targeting the Baliao (Shangliao, Ciliao, Zhongliao, Xialiao) and Ashi points (local tender spots), supplemented by daily Five-Form Tai Chi exercises. Acupuncture sessions were conducted once daily, with needles retained for 10 minutes, over a consecutive period of 14 days. Furthermore, the Tai Chi exercises were performed twice daily, with each session lasting 20 minutes, also spanning a duration of 14 days.

**Outcomes:**

Post-treatment, the Visual Analog Scale pain score decreased from 7 to 3. Follow-up computed tomography revealed no obvious fractures on sacrococcygeal plain scans, with recommendations for follow-up or magnetic resonance imaging if necessary. The patient showed significant functional improvement and reduced anxiety.

**Lessons:**

Acupuncture combined with Tai Chi effectively alleviates pain, promotes functional recovery, and accelerates fracture healing in patients with coccyx fractures and subluxations.

## 1. Introduction

Coccyx fractures and subluxations are common orthopedic injuries typically caused by direct trauma such as falls or impacts. Clinically, they manifest as sacrococcygeal pain and difficulty sitting or standing.^[1]^ Based on the large-scale study of the Korean National Claims Database (2010–2018), the annual incidence rate of tailbone fractures in 2018 was 119.75/100,000 people. Among them, the male incidence rate is 33.44/100,000, and the female 86.30/100,000, with a ratio of 1:2.6 (significantly higher for women).^[2]^ Etiologies include direct trauma, falls, ligament strains, chronic inflammation, nerve damage, and congenital coccygeal anomalies (e.g., hook-shaped coccyx), which may lead to pain and functional impairment.^[3,4]^ Current conservative treatments often involve transanal manual reduction, while surgical interventions (e.g., coccygectomy) are reserved for refractory cases.^[5]^ Modern medical management emphasizes bed rest and manual reduction, but some patients experience slow recovery, chronic pain, emotional distress, or reluctance to undergo transanal procedures. Acupuncture may alleviate pain and relieve local muscle spasms,^[6,7]^ while Tai Chi, a traditional Chinese exercise, enhances perianal muscle stability and reduces anxiety.^[8]^ This case report explores the combined use of acupuncture and Tai Chi for coccyx fracture rehabilitation, offering insights into integrative Chinese-Western medicine.

## 2. Case report

### 2.1. Patient information

**Figure 1.**
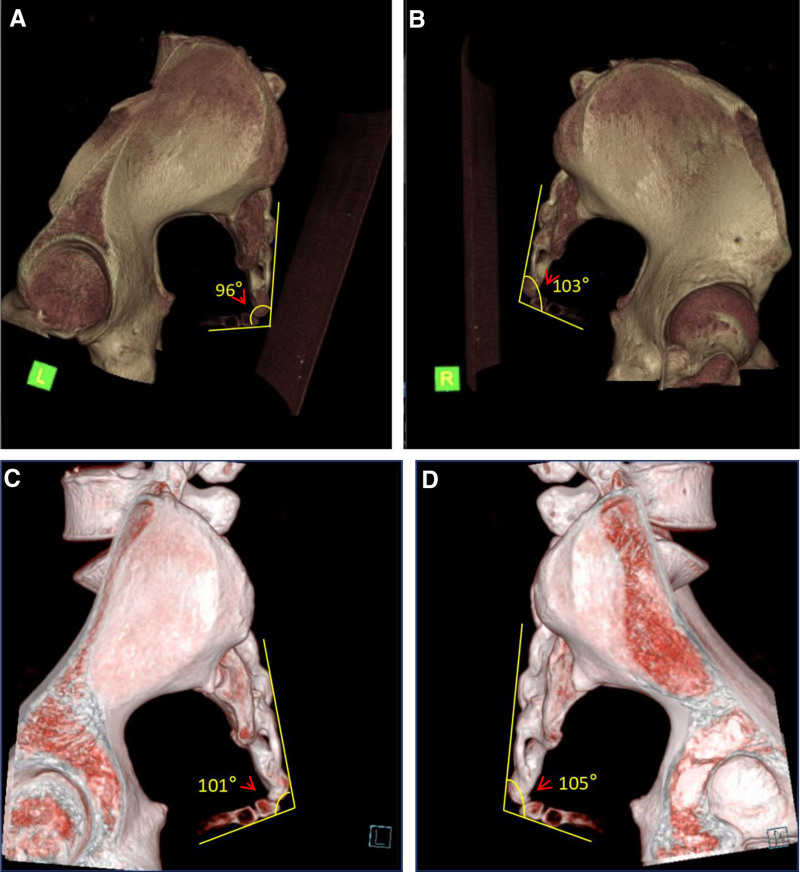
Pretreatment and posttreatment CT 3D reconstruction. (A) Pretreatment CT 3D reconstruction (February 13, 2025); (B) posttreatment CT 3D reconstruction (March 20, 2025). CT = computed tomography.

A 34-year-old female patient sustained a fall while skiing on February 13, 2025, landing on her buttocks, which resulted in immediate severe sacrococcygeal pain and an inability to sit for prolonged periods. Computed tomography (CT) findings at the local hospital included: a suspected fracture of the S5 vertebra (evidenced by a lucent line); a possible subluxation of the Co1 vertebra; swelling and effusion of the sacrococcygeal soft tissues (Fig. 1, refer to Fig. 1A: pretreatment CT 3D reconstruction).

### 2.2. Treatment protocol

The patient underwent acupuncture treatment with deep needling at predetermined acupoints, using disposable stainless steel needles measuring 0.25 mm in diameter and 45 mm in length, manufactured by Suzhou Medical Products Factory Co., Ltd., Suzhou, China.

**Figure 2.**
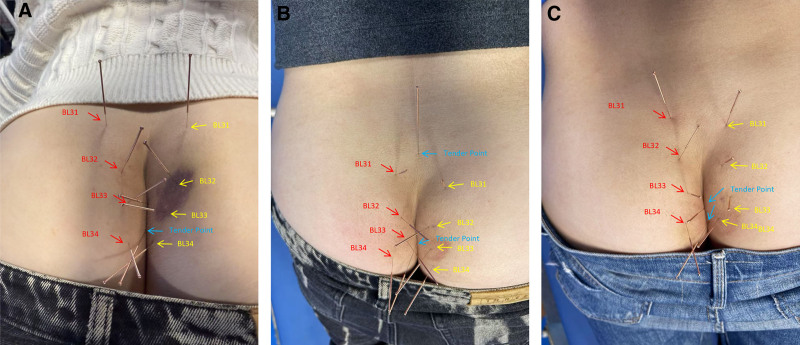
Acupuncture treatment and acupoint selection. (A) First acupuncture session; (B) 7th acupuncture session; and (C) 14th acupuncture session. Acupoints: right Shangliao (BL31): needle insertion depth of 2 cm. Left Shangliao (BL31): needle insertion depth of 2 cm. Right Ciliao (BL32): needle insertion depth of 2 cm. Left Ciliao (BL32): needle insertion depth of 2 cm. Right Zhongliao (BL33): needle insertion depth of 2 cm. Left Zhongliao (BL33): needle insertion depth of 2 cm. Right Xialiao (BL34): needle insertion depth of 2 cm. Left Xialiao (BL34): needle insertion depth of 2 cm.

The acupoints prescription is as follows: Ashi acupoints (local tender spots), Shangliao (BL31), Ciliao (BL32), Zhongliao (BL33), and Xialiao (BL34), employing a uniform reinforcing-reducing technique. Needles were retained for 10 minutes daily for 14 days (Fig. 2).

### 2.3. Tai Chi exercise

**Figure 3.**
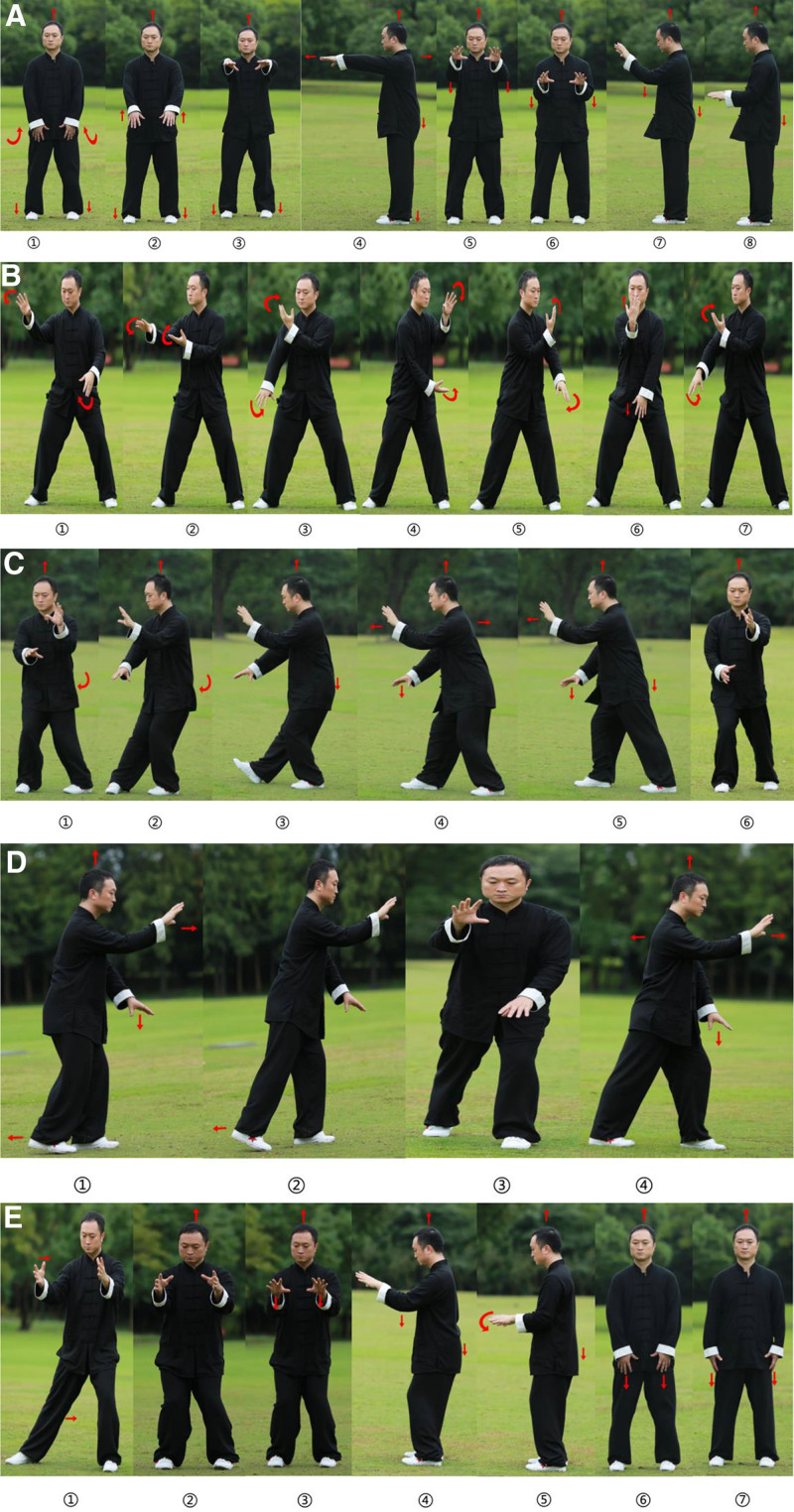
Illustration of 5 Tai Chi movements. (A) Commencement form; (b) cloud hands; (C) brush knee and twist step; (D) step back and repulse monkey; and (E) closing form. Ashi points: Ah Shi Point (Āshìxué) refers to non-meridian, palpation-located tender points identified through physical examination where pressure elicits pain or discomfort. These points lack fixed anatomical positions but dynamically manifest in areas of myofascial dysfunction, serving as therapeutic targets for needling or manual therapy. Baliao points(BL31–BL34): refers to a group of 4 bilateral acupuncture points (totaling 8 points) located over the 4 posterior sacral foramina. These points are clinically significant for regulating the lower abdomen, pelvic organs, and lumbar-sacral region. Their nomenclature derives from the anatomical structure: “Ba” (8) denotes the total number of points, while “Liao” (crevice) describes their position in the osseous depressions of the sacrum.

Five-form simplified Tai Chi (commencement form; cloud hands; brush knee and twist step; step back and repulse monkey; and closing form), emphasizing gentle lumbosacral rotation and weight shifting. Performed twice daily (20 minutes/session) for 14 days (Fig. 3).

### 2.4. Quality control

The Tai Chi instructor held a PhD in Acupuncture and Tuina, with postdoctoral training at the University of Ottawa School of Human Kinetics and Shanghai University of Sport. As a 6th-generation lineage holder of Wu-style Tai Chi, he supervised the intervention. The patient maintained excellent adherence, verified through daily practice logs.

### 2.5. Outcomes

#### 2.5.1. Symptom improvement

By day 7, the Visual Analog Scale score decreased from 7 to 5, with prolonged sitting tolerance. By day 14, the Visual Analog Scale dropped to 3, and daily activities resumed without restriction.

#### 2.5.2. Imaging follow-up

A repeat CT on March 20, 2025, showed no evident fractures on sacrococcygeal plain scans, recommending a follow-up or MRI if necessary (Fig. 1, refer to Fig. 1B posttreatment CT 3D reconstruction).

#### 2.5.3. Follow-up

At 1 month posttreatment, the patient reported no pain recurrence, only mild soreness after prolonged sitting, and continued Tai Chi practice.

## 3. Discussion

Studies indicate that non-displaced fractures can often be treated conservatively. However, the presence of skin dimpling, also known as the pucker sign, should raise concerns about soft tissue interposition. After emergency reduction and immobilization, it is recommended to refer the patient to an orthopedic specialist. In this case, the patient’s coccygeal fracture was presented without skin dimpling but with localized ecchymosis only.^[9–11]^

In this case, acupuncture at Baliao points (located near the sacral plexus) and Ashi points alleviated pain, reduced muscle spasms, and promoted inflammatory absorption. Baliao points correspond anatomically to the sacral nerve posterior branches, and stimulation may modulate the neuro-immune-endocrine network, suppress pro-inflammatory cytokines (e.g., IL-6 and TNF-α), and enhance β-endorphin secretion for analgesia.^[12]^ Ashi points directly target injured areas, improving microcirculation and hematoma resolution. Additionally, acupuncture activates motor cortex and pain-modulating brain regions (e.g., insula and anterior cingulate cortex), mitigating chronic pain via central sensitization.^[13]^

The slow movements of Tai Chi emphasize coordinated movements of the lumbar and sacral regions, which can enhance pelvic floor muscle strength and improve stability after coccyx fractures. Clinical studies have shown that Tai Chi training can alleviate chronic back pain (including sacrococcygeal pain), and its mechanism may be related to enhancing core muscle groups and improving posture balance. Therefore, this study selected Tai Chi to exercise the pelvic floor muscles and lumbar sacral coordination of patients.

Baliao points (BL31–BL34) are essential acupoints for treating sacrococcygeal and lumbar pain. They demonstrate particular efficacy in managing postfracture pain and dysfunction (e.g., pelvic floor muscle spasms and bowel/bladder irregularities) following coccygeal injuries. The slow movements of Tai Chi emphasize lumbosacral coordination, strengthening pelvic floor musculature and enhancing postfracture stability. Clinical studies confirm that Tai Chi alleviates chronic low back pain (including sacrococcygeal pain), potentially through improved core muscle activation and postural balance.^[14]^

Tai Chi slow, controlled movements strengthen the lumbosacral multifidus, erector spinae, and pelvic floor muscles, enhancing spinal-pelvic stability and reducing coccygeal stress. Biomechanically, Tai Chi optimizes weight distribution, minimizing shear forces at the sacrococcygeal joint to correct subluxation-induced imbalance.^[15]^ This case demonstrated not only clinical improvement but also radiological resolution, suggesting that combined therapy may achieve compensatory repair through soft tissue biomechanical regulation.

## 4. Conclusion

This case highlights the efficacy of combining acupuncture with Tai Chi for alleviating pain, restoring function, and accelerating recovery from coccyx fractures and subluxations, offering an alternative to surgical intervention. In clinical practice, this integrated approach should be tailored to individual pain tolerance, muscle conditions, and imaging findings. It is crucial to acknowledge that this study has its limitations, such as a follow-up period of only 1 month, which is deemed insufficient for evaluating fracture healing and long-term functional outcomes. Therefore, the study suggests enrolling patients in a long-term follow-up program to track their recovery over time. Future research should focus on elucidating the molecular and biomechanical mechanisms that underpin the integration of Chinese and Western therapies to improve evidence-based precision rehabilitation.

## Acknowledgments

We would like to express our gratitude to the patients for granting permission to use their clinical data in this paper and for the publication of this research.

## Author contributions

**Conceptualization:** Junchang Liu, Qingguang Zhu.

**Investigation:** Yukui Tian, Lei Guo.

**Supervision:** Jingxian Li, Junchang Liu, Qingguang Zhu.

**Visualization:** Cheng Wang, Lingjun Kong.

**Writing – original draft:** Xue Bai.

**Writing – review & editing:** Xue Bai.
